# The Protein Phosphatase PPM1G Destabilizes HIF-1α Expression

**DOI:** 10.3390/ijms19082297

**Published:** 2018-08-05

**Authors:** Jaehyuk Pyo, Jaewook Ryu, Wootae Kim, Jae-Sun Choi, Joo-Won Jeong, Ja-Eun Kim

**Affiliations:** 1 Department of Biomedical Science, Graduate School, Kyung Hee University, Seoul 02447, Korea; biopyo37@naver.com (J.P.); busterray@naver.com (J.R.); mubear@khu.ac.kr (W.K.); jaesun0505@naver.com (J.-S.C.); jjeong@khu.ac.kr (J.-W.J.); 2 Department of Anatomy and Neurobiology, School of Medicine, Kyung Hee University, Seoul 02447, Korea; 3 Department of Pharmacology, School of Medicine, Kyung Hee University, Seoul 02447, Korea

**Keywords:** HIF-1α, PPM1G, hypoxia, normoxia

## Abstract

Hypoxia-inducible factors (HIFs) are key regulators of hypoxic responses, and their stability and transcriptional activity are controlled by several kinases. However, the regulation of HIF by protein phosphatases has not been thoroughly investigated. Here, we found that overexpression of Mg^2+^/Mn^2+^-dependent protein phosphatase 1 gamma (PPM1G), one of Ser/Thr protein phosphatases, downregulated protein expression of ectopic HIF-1α under normoxic or acute hypoxic conditions. In addition, the deficiency of PPM1G upregulated protein expression of endogenous HIF-1α under normoxic or acute oxidative stress conditions. PPM1G decreased expression of HIF-1α via the proteasomal pathway. PPM1G-mediated HIF-1α degradation was dependent on prolyl hydroxylase (PHD), but independent of von Hippel-Lindau (VHL). These data suggest that PPM1G is critical for the control of HIF-1α-dependent responses.

## 1. Introduction

Hypoxia-inducible factors (HIFs), members of the bHLH/PAS (basic helix-loop-helix/PER-ARNT-SIM) family, are transcription factors that mainly respond to oxygen deprivation [1]. HIFs are heterodimeric complexes composed of a hypoxia-inducible α subunit and a constitutively expressed β subunit. There are at least three α subunits, namely, HIF-1α, HIF-2α, and HIF-3α, and one β subunit, also known as ARNT (aryl hydrocarbon receptor nuclear translocator) [2]. The HIF dimer binds to hypoxia response elements (HREs) with the consensus sequence RCGTG (where R is either A or G) [3] and transactivates hundreds of genes that encode proteins involved in stem cell renewal, angiogenesis, erythropoiesis, metabolism, metastasis, cell proliferation, and cell survival [4,5]. Therefore, HIFs help to regulate several physiological and pathological responses [6].

The most well-studied α subunit is HIF-1α. Expression of HIF-1α is controlled by transcription, translation, chaperone binding, and post-translational modifications (PTMs). Whereas HIF-1α is constitutively transcribed and translated under normoxia, the half-life of HIF-1α protein is less than 5 min [7]. The continuous degradation of HIF-1α is promoted via O_2_-dependent proline hydroxylation by prolyl-4-hydroxylase domain-containing proteins (PHDs) and subsequent ubiquitination by the von Hippel-Lindau (VHL)-containing E3 ubiquitin ligase complex [8,9]. Other O_2_-independent PTMs also affect the stability of HIF-1α, in addition to hydroxylation. SUMOylation of HIF-1α regulates its stability, although the effect of SUMOylation on HIF-1α degradation is very controversial [10,11,12,13,14]. HIF-1α is stabilized following acetylation by p300 [15], but destabilized following acetylation by ARD1 [16]. SET7/9-mediated methylation of HIF-1α decreases its stability [17]. The effect of phosphorylation on the stability of HIF-1α depends on the kinase involved [18,19]; it has been reported that HIF-1α is destabilized by GSK-3β [20] and PLK3 [21], but stabilized by CDK1 [22] and ATM [23]. The reversal of these PTMs, such as deubiquitination [24], deSUMOylation [25], deacetylation [26,27], and demethylation [17], also regulates HIF-1α stability. As for phosphatase regulating HIF-1α, SHP-1 decreases HIF-1α’s expression although direct dephosphorylation of HIF-1α by SHP-1 has not been determined [28]. MKP-1/DUSP1 inhibits HIF-1α’s activity through inactivating ERK [29]. While the phosphorylation of HIF-1α by several kinases has been extensively investigated, its direct dephosphorylation by phosphatases is less understood.

Members of the protein phosphatase (PP) family include enzymes that dephosphorylate tyrosine and serine/threonine. Serine/threonine phosphatases are divided into the following subfamilies: phosphoprotein phosphatases (PPPs), including PP1, PP2A, PP2B, PP4, PP5, and PP6; metal-dependent protein phosphatases (PPMs), including PP2C and pyruvate dehydrogenase phosphatase; and aspartate-based phosphatases such as FCP/SCP [30]. PPMs are Mg^2+^- or Mn^2+^-dependent single-subunit proteins, while PPPs are multi-subunit complexes. PPM subfamily includes PP2Cα, PP2Cβ, PP2Cγ, PP2Cδ (also known as Wip1 or PPM1D), and PH domain leucine-rich repeat protein phosphatase [31]. PP2Cγ is now commonly called Mg^2+^/Mn^2+^-dependent protein phosphatase 1 gamma (PPM1G). This PP regulates multiple cellular functions; the phosphatase activity of PPM1G is required for spliceosome assembly [32,33], localization of the SMN complex to Cajal bodies [34], promotion of gene-specific transcription [35,36], inhibition of 4E-BP1-mediated cap-dependent translation [37,38], regulation of the G1-S cell cycle transition [39,40], histone chaperoning for H2A-H2B exchange [41], involvement in the DNA damage response [42,43], and cell survival and neural development [44]. Other functions of PPM1G have not been thoroughly studied.

While phosphorylation of HIF-1α and its regulatory proteins has been well studied, the dephosphorylation in the regulation of HIF-1α and the HIF-1α-related pathway is less understood. Here, we examined the effect of PPM1G on HIF-1α expression and found that PPM1G negatively regulates HIF-1α.

## 2. Results

### 2.1. Hypoxia-Inducible Factor (HIF)-1α Expression Is Negatively Regulated by Protein Phosphatase 1 Gamma (PPM1G)

**Figure 1:**
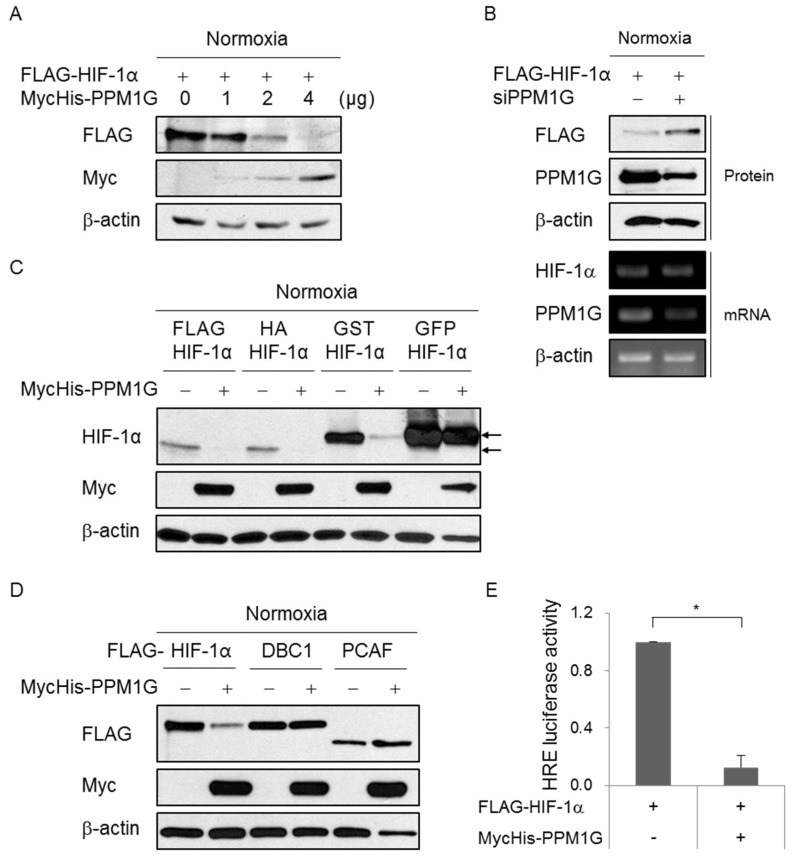
Protein phosphatase 1 gamma (PPM1G) downregulates hypoxia-inducible factor (HIF)-1α expression under normoxic conditions. HEK293T cells were transfected with MycHis-Empty (−) or MycHis-PPM1G (+) (**A**), control siRNA (−) or PPM1G-targeting siRNA (siPPM1G) (**B**), vectors harboring HIF-1α fused with various tags (**C**), and vectors encoding other proteins (**D**). Cells were then cultured under normoxic conditions. Expression of each protein was determined by Western blotting and PPM1G knockdown was confirmed by RT-PCR. (**A**) FLAG-HIF-1α expression in cells transfected with various amounts of the PPM1G vector; (**B**) Expressions of FLAG-HIF-1α protein and PPM1G mRNA in PPM1G-depleted cells; (**C**) FLAG-HIF-1α, HA-HIF-1α, GST-HIF-1α, and GFP-HIF-1α expression in PPM1G-overexpressing cells. Arrows indicate tagged HIF-1α; (**D**) FLAG-HIF-1α, FLAG-DBC1, and FLAG-PCAF expression in PPM1G-overexpressing cells; (**E**) HEK293T cells were co-transfected with the PGK1-HRE-Luc reporter, a β-galactosidase-encoding plasmid and either MycHis-Empty (−) or MycHis-PPM1G (+) in the presence of HIF-1α overexpression. Relative luciferase activity was calculated after normalization of transfection efficiency according to the β-galactosidase activity. * *p* < 0.05; significantly different from only FLAG-HIF-1α-transfected cells.

To determine whether PPM1G regulates HIFs, HIF-1α expression was monitored following PPM1G overexpression. Under normoxic conditions, overexpression of PPM1G significantly reduced ectopic HIF-1α expression in a PPM1G dose-dependent manner (Figure 1A). By contrast, knockdown of PPM1G enhanced ectopic HIF-1α expression (Figure 1B). The knockdown efficiency of PPM1G was verified by reverse transcription-polymerase chain reaction (RT-PCR) (Figure 1B). Next, to determine whether PPM1G specifically inhibits HIF-1α expression, cells were transfected with constructs encoding HIF-1α fused with various tags or other FLAG-tagged proteins. PPM1G downregulated all the tagged HIF-1α proteins (Figure 1C), indicating that the PPM1G-mediated HIF-1α downregulation is not tag protein-specific. By contrast, PPM1G did not downregulate expressions of CCAR2 (cell cycle and apoptosis regulator 2) or PCAF (p300/CBP-associated factor) used as a negative control which possess the same tag but has no relevance with PPM1G, suggesting that PPM1G does not promote general downregulation of proteins (Figure 1D). The data demonstrate that PPM1G specifically downregulates HIF-1α expression in normoxia. Downregulation of HIF-1α expression might reduce the transcriptional activity of HIF. To investigate this, HIF transcriptional activity was measured in PPM1G-overexpressing cells using an HRE-containing luciferase reporter gene. As expected, PPM1G reduced HRE-luciferase activity under normoxic conditions (Figure 1E). Overall, this indicates that PPM1G inhibits HIF-1α expression and its transcriptional activity.

### 2.2. HIF-1α Is Downregulated by PPM1G in Normoxia and Upon Acute Hypoxic and Oxidative Stress

**Figure 2:**
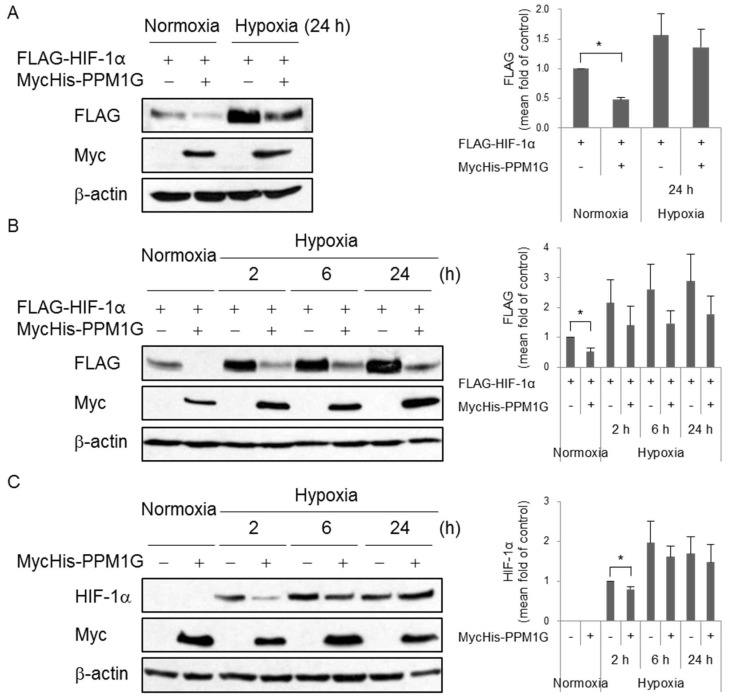

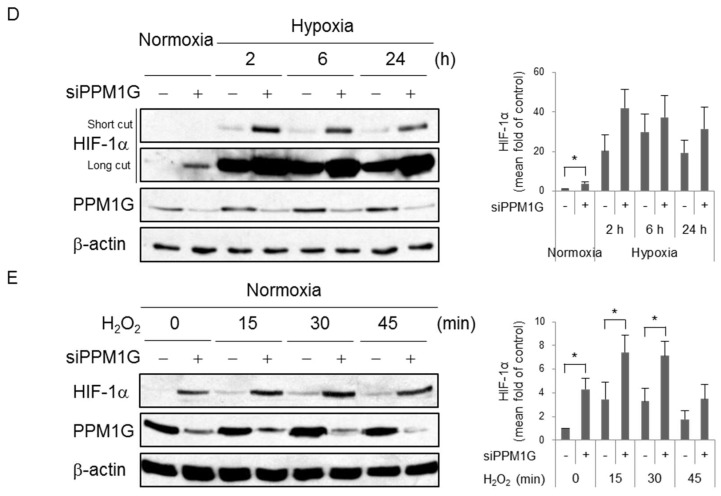
PPM1G affects HIF-1α expression under hypoxic and oxidative stress conditions. (**A**–**E**) HEK293T cells were co-transfected with FLAG-HIF-1α, MycHis-Empty (−), MycHis-PPM1G (+), control siRNA (−) or PPM1G-targeting siRNA (siPPM1G). The HEK293T cells were exposed to normoxia or hypoxia (**A**–**D**), or 0.5 mM H_2_O_2_ (**E**) for the indicated durations. Expression of ectopic FLAG-HIF-1α (**A**,**B**) or endogenous HIF-1α (**C**–**E**) was determined by Western blotting. * *p* < 0.05; significantly different from the matched control group.

HIF-1α is an integral factor in response to hypoxia. To determine whether PPM1G controls HIF-1α expression upon hypoxic stress, cells were exposed to hypoxia following overexpression or knockdown of PPM1G. As shown in Figure 1, overexpression of PPM1G significantly downregulated ectopic HIF-1α expression under normoxic conditions (lane 1 vs. lane 2, Figure 2A). This effect was also observed, but not significantly, under hypoxic conditions (lane 3 vs. lane 4, Figure 2A). To determine the effect of hypoxia on PPM1G-mediated HIF-1α downregulation, cells were exposed to hypoxia for various durations. The downregulation of ectopic HIF-1α by PPM1G also occurred as the duration of hypoxia increased although its effect was not statistically significant (Figure 2B). The effect of PPM1G on endogenous HIF-1α was also tested. Overexpression of PPM1G slightly reduced endogenous HIF-1α expression under acute hypoxic conditions (2 h), but not under prolonged hypoxic conditions (6 or 24 h) (Figure 2C); the different effects of overexpressed PPM1G on ectopic and endogenous HIF-1α in acute hypoxia (2 h) might be due to antibodies such as anti-FLAG and anti-HIF-1α which detect HIF-1α only in transfected cells and in both non-transfected- and transfected cells, respectively. Next, the effect of PPM1G on endogenous HIF-1α was evaluated in PPM1G siRNA-transfected cells. Knockdown of PPM1G significantly increased HIF-1α expression under normoxic conditions. The negative effect of PPM1G on HIF-1α expression also occurred under hypoxic conditions although it was not statistically significant (Figure 2D). In addition, we also verified the effect of PPM1G on HIF-1α expression in other stress conditions; H_2_O_2_ treatment induces HIF-1α upregulation [45]. PPM1G-deficient cells showed higher expression of HIF-1α following oxidative stress (Figure 2E). The differential HIF-1α expression between control siRNA- and PPM1G siRNA-transfected cells was attenuated as duration of H_2_O_2_ treatment increased. However, there still is a tendency for PPM1G to negatively regulate HIF-1α expression. Overall, PPM1G negatively regulates HIF-1α expression in normal and stress conditions.

### 2.3. PPM1G Promotes HIF-1α Degradation via the Proteasomal Pathway

**Figure 3:**
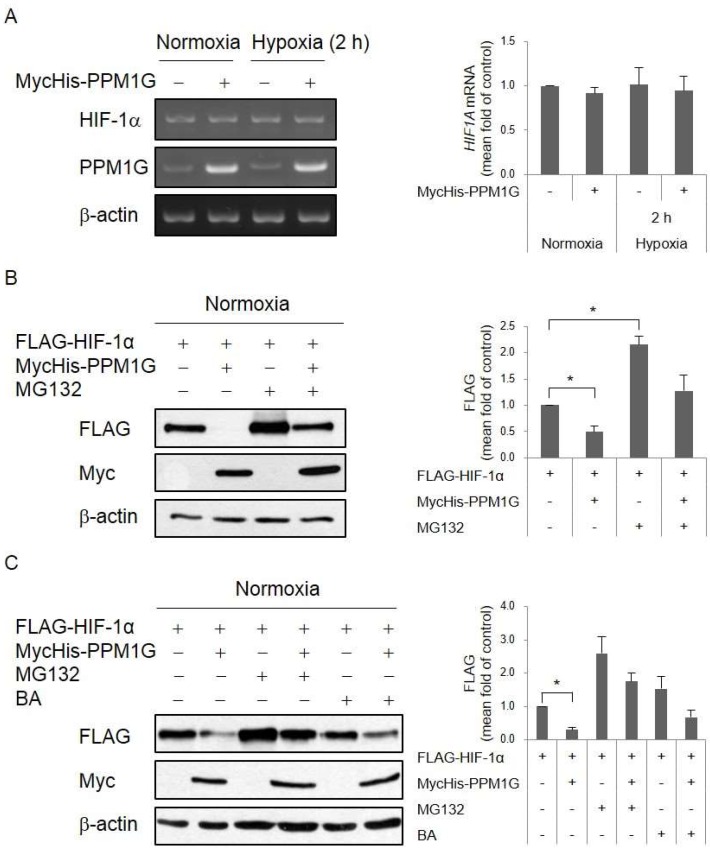
PPM1G induces proteasomal degradation of HIF-1α. HEK293T cells were co-transfected with FLAG-HIF-1α and MycHis-Empty (−) or MycHis-PPM1G (+), and exposed to normoxia or hypoxia. (**A**) mRNA expressions of the HIF-1α and β-actin were measured by RT-PCR; (**B**,**C**) Expression of each protein was determined by Western blotting. (**B**) Proteasome-mediated protein degradation was blocked by treating cells with 10 µM MG132 for 6 h prior to lysis; (**C**) Proteasome- and lysosome-mediated protein degradation was blocked by treating cells with 10 µM MG132 and 10 nM bafilomycin A1 (BA) (**A**,**B**) for 12 h prior to lysis. * *p* < 0.05; significantly different from the matched control group.

We next sought to elucidate how PPM1G regulates HIF-1α expression. First, expression of *HIF1A* gene was determined by RT-PCR. However, the results showed that the expression of HIF-1α mRNA was not changed by PPM1G overexpression (Figure 3A). It suggests that PPM1G does not decrease transcriptional or post-transcriptional level of *HIF1A* gene. Next, we prompted to check whether PPM1G affects post-translational level of HIF-1α. HIF-1α is degraded via the proline hydroxylation-dependent proteasomal pathway in normoxia [8,9]. To block proteasome-mediated degradation, cells were treated with MG132, an inhibitor of the 26S proteasome. MG132 treatment recovered PPM1G-mediated downregulation of HIF-1α protein expression (Figure 3B). HIF-1α also undergoes lysosome-mediated degradation via chaperone-mediated autophagy [46,47]. Treatment with bafilomycin A1, an inhibitor of autophagosome-lysosome fusion via vacuolar-type H(+)-ATPase-dependent acidification, slightly recovered PPM1G-mediated downregulation of HIF-1α protein expression (Figure 3C). However, the recovery ratio in bafilomycin A1-treated cells was not as high as in MG132-treated cells. This demonstrates that PPM1G induces degradation of HIF-1α protein partially via the lysosomal pathway, but mainly via the proteasomal pathway.

### 2.4. PPM1G Promotes HIF-1α Degradation in a PHD-Dependent Manner

**Figure 4:**
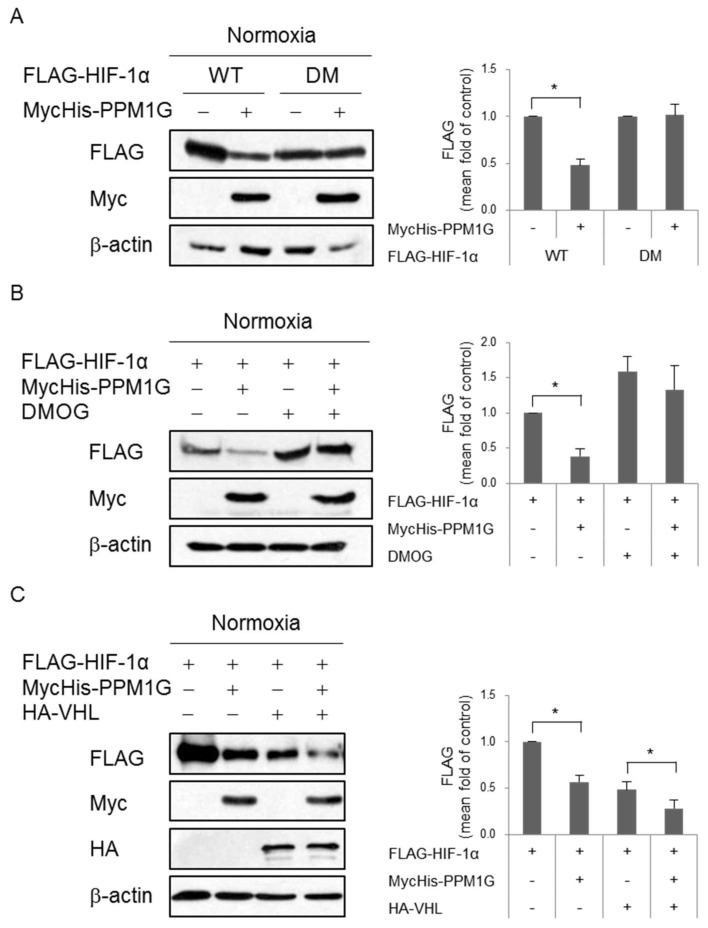
PPM1G reduces HIF-1α expression in a PHD-dependent, but VHL-independent, manner. Cells were transfected with the indicated vectors. Expression of each protein was determined by Western blotting. (**A**) HEK293T cells were co-transfected with MycHis-empty (−) or MycHis-PPM1G (+) and FLAG-HIF-1α-wild type (WT) or -P402A/P564A double mutant (DM); (**B**) PHD activity was blocked by treating the transfected HEK293T cells with 1 mM dimethyloxaloylglycine (DMOG) for 12 h prior to lysis; (**C**) 786-O cells, which do not express VHL and HIF-1α, were co-transfected with FLAG-HIF-1α, MycHis-PPM1G, and HA-VHL, and then exposed to normoxia. * *p* < 0.05; significantly different from the matched control group.

The mechanism underlying proteasomal degradation of HIF-1α has been thoroughly studied. HIF-1α degradation is primarily mediated by PHDs such as PHD1, PHD2, and PHD3, but mainly by PHD2 [48]. PHD-mediated hydroxylation of HIF-1α at Pro402 and Pro564 within the oxygen-dependent degradation domain enhances its binding to the VHL-containing E3 ubiquitin ligase complex [49]. Subsequent ubiquitination of HIF-1α promotes its degradation via the 26S proteasome [8]. To determine whether PPM1G-dependent proteolysis of HIF-1α is affected by its proline hydroxylation, a HIF-1α mutant (DM) with the P402A/P564A double mutation was used. PPM1G significantly decreased expression of HIF-1α-wild type (WT), but did not affect that of DM (Figure 4A). This suggests that hydroxylation of HIF-1α is required for its PPM1G-mediated degradation. Next, to confirm that PHD-dependent hydroxylation of HIF-1α is necessary for its PPM1G-mediated degradation, cells were treated with dimethyloxaloylglycine (DMOG), a competitive inhibitor of PHDs. PPM1G did not downregulate HIF-1α expression in the presence of DMOG (Figure 4B). Overall, these data suggest that PPM1G promotes proline hydroxylation-dependent degradation of HIF-1α. We next investigated whether PHD-dependent HIF-1α degradation occurs in a VHL-dependent manner. 786-O cells, which do not express VHL and HIF-1α, were transfected with both genes to determine whether degradation of HIF-1α is dependent on VHL. Under normoxic conditions, PPM1G downregulated HIF-1α expression in the absence (lane 1 vs. lane 2) and presence (lane 3 vs. lane 4) of VHL (Figure 4C), suggesting that VHL does not mediate PPM1G-dependent HIF-1α degradation. This indicates that PPM1G induces HIF-1α degradation in a VHL-independent manner, although the mechanism of proteasomal degradation of HIF-1α further needs to be unveiled. Overall, our results demonstrate that proline hydroxylation of HIF-1α is indispensable for its PPM1G-mediated proteasomal degradation.

## 3. Discussion

PPM1G downregulated HIF-1α expression via the proteasomal pathway and reduced the transcriptional activity of HIF in normoxia. The inhibitory effect of PPM1G on HIF-1α expression also occurred under stress conditions such as acute hypoxia and oxidative stress. This indicates that PPM1G is one of the factors that promote HIF-1α degradation. The stability of HIF-1α is regulated via several PTMs. As described in the Introduction section, regulation of HIF-1α by ubiquitination, SUMOylation, acetylation, and phosphorylation has been thoroughly studied. By contrast, the roles of the reverse PTMs (e.g., dephosphorylation) in the regulation of HIF-1α and/or its regulatory factors are less understood. Whereas there are no studies about the direct dephosphorylation of HIF-1α even by other PP family proteins, a recent report showed that PP2A dephosphorylates PHD2, a HIF-1α regulatory factor [50]. The B55α regulatory subunit of PP2A interacts with PHD2, and then PP2A dephosphorylates PHD2 at S125. Dephosphorylated PHD2 fails to hydroxylate HIF-1α, resulting in stabilization of HIF-1α. Considering the relationship between PPs and PHDs, the binding of PPM1G to PHDs should be further investigated.

This study showed that PPM1G induced degradation of HIF-1α in a PHD-dependent and VHL-independent manner. In general, proline-hydroxylated HIF-1α binds to the VHL-containing E3 ubiquitin ligase complex composed of elongin B, elongin C, cullin-2, and the small RING finger protein RBX [51]. Thereafter, ubiquitinated HIF-1α is degraded by the proteasome. However, PPM1G induced proteasomal degradation of HIF-1α independently of VHL. In our experiments, we confirmed the negative effect of PPM1G on expression of both endogenous and ectopic HIF-1α. However, there might be an additional system to degrade overexpressed HIF-1α in normoxia when cells were transfected with ectopic HIF-1α. Except for VHL, there would be an additional regulatory system to control HIF-1α expression in PPM1G-dependent manner. Similarly, methylselenocysteine treatment induces degradation of HIF-1α in a PHD2-dependent, but VHL-independent, manner [52]. Further studies are required to identify which E3 ubiquitin ligase is responsible for PPM1G-dependent HIF-1α degradation. In addition, PPM1G may modulate the activities of deubiquitinases that help to control HIF-1α stability, such as VHL protein-interacting deubiquitinating enzyme 2 (VDU2) [53], ubiquitin-specific protease 8 (USP8) [54], OUT domain-containing protein 7B (OTUD7B) [55], ubiquitin C-terminal hydrolase-L1 (UCHL1) [56], and HAUSP [57]. PPM1G is not reported to be related to any such deubiquitinase except for HAUSP. In fact, USP7S, an isoform of HAUSP, is downregulated and inactivated via dephosphorylation by PPM1G [43]. Low expression and inactivity of USP7S lead to ubiquitination and proteasomal degradation of Mdm2, which stabilizes p53. It would be interesting to investigate whether dephosphorylation of HAUSP by PPM1G affects its deubiquitinase activity and thereby regulates HIF-1α stability.

The inhibition of HIF-1α expression by PPM1G occurs following several cellular insults such as hypoxia and oxidative stress. Based on our findings, PPM1G could be critical for the regulation of HIF-1α-dependent cellular responses in following cellular conditions. Mutation or promoter hypermethylation of VHL in clear-cell renal carcinoma would give rise to the upregulation of HIF-1α. The responses to anti-cancer therapy using doxorubicin and ionizing radiation would be diminished because of HIF-1α upregulation [58,59]. HIF-1α is upregulated by non-steroidal anti-inflammatory drugs such as acetylsalicylic acid and naproxen [60,61]. HIF-1α expression is also induced by cytokines and microbes in inflammatory and infectious conditions, respectively, for a host defense mechanism [62,63]. PPM1G may be a useful target to regulate HIF-1α-dependent responses by controlling HIF-1α protein expression in several physiological and pathological conditions.

## 4. Materials and Methods

### 4.1. Chemical Compounds

Polyethylenimine (PEI, 408727) and bafilomycin A1 (BA, B1793) was obtained from Sigma (St. Louis, MI, USA). MG132 (10012628), and dimethyloxaloylglycine (DMOG, D1070) was purchased from Cayman (Ann Arbor, MI, USA), and Frontier Scientific (Logan, UT, USA), respectively. Hydrogen peroxide (H_2_O_2_, E882) was purchased from Amresco Biochemicals (Solon, OH, USA).

### 4.2. Cell Culture, Hypoxia Condition and Treatment

HEK293T embryonic kidney cells and 786-O renal adenocarcinoma cells were maintained in Dulbecco’s modified Eagle’s medium supplemented with 10% fetal bovine serum, 100 U/mL penicillin G sodium, 100 µg/mL streptomycin sulfate, and 0.25 µg/mL amphotericin B. Cells were exposed to 21% O_2_ (normoxia) or 1% O_2_ (hypoxia). Hypoxic conditions were achieved by placing cell plates in a humidified tightly sealed chamber (27310, STEMCELL Technologies Inc., Vancouver, BC, Canada) or hypoxic workstation (INVIVO2, Ruskinn Technology, Bridgend, UK) with triple gas mixture of 1% O_2_, 5% CO_2_, and 94% N_2_.

### 4.3. Plasmid Transfection

Cells were transfected with each construct using PEI. Six hours after transfection, the media were replaced with fresh complete media. The cells were applied for each analysis 48 h after transfection.

### 4.4. Small Interfering RNA (siRNA) Transfection

Control and PPM1G siRNA were synthesized by ST Pharm. Co., Ltd. (Seoul, Korea) and Dharmacon (Lafayette, CO, USA), respectively. The siRNA duplexes were as follows: control siRNA sense strand, AUGAACGUGAAUUGCUCAAdTdT; PPM1G siRNA sense strand, GAGCAGCCAGGAAGUUGUAdTdT. Cells were transfected with 20 nM siRNA using Lipofectamine RNAiMax (13778-150, Invitrogen, Carlsbad, CA, USA). The cells were applied for each analysis 48 h after transfection.

### 4.5. Preparation of Crude Cell Extract and Western Blotting

Cells were lysed on ice for 10 min using NETN lysis buffer (100 mM NaCl, 1 mM EDTA, 20 mM Tris-HCl, 0.5% Nonidet P-40, 50 mM β-glycerophosphate, 10 mM NaF, and 1 mM Na_3_VO_4_) containing a protease inhibitor cocktail (535140, Millipore, Burlington, MA, USA). After centrifugation at 12,000× *g* for 5 min, the supernatant was saved as a crude cell extract. This was boiled in Laemmli buffer and loaded onto a sodium dodecyl sulfate (SDS)-polyacrylamide gel. Western blotting was performed according to a standard protocol. The following antibodies were used for Western blotting: FLAG (F3165, Sigma, St. Louis, MO, USA), HA (MMS-101R, Covance, Princeton, NJ, USA), Myc (sc-40, Santa Cruz Biotechnology, Dallas, TX, USA), HIF-1α (610958, BD Biosciences, Franklin Lakes, NJ, USA), PPM1G (ab70794, Abcam, Cambridge, UK, USA), and β-actin (4970, Cell Signaling, Danvers, MA, USA).

### 4.6. Reverse Transcription-Polymerase Chain Reaction (RT-PCR)

Total RNA was isolated using Trizol reagent (Invitrogen, Carlsbad, CA, USA) and was used to synthesize cDNA using PrimeScript^TM^ reverse transcriptase (Takara, Kusatsu, Shiga, Japan). Synthesized cDNA was amplified, and the PCR product was then visualized on 1% agarose gel. The sequences of each forward (F) and reverse (R) primer used for PCR were as follows: HIF-1α-F, CAGAAGATACAAGTAGCCTC; HIF-1α-R, CTGCTGGAATACTGTAACTG; PPM1G-F, GACCACTGAAGAAGTCATTA; PPM1G-R, CAGAGGCTGAAGAGCAGG; β-actin-F, GCTCGTCGTCGACAACGGCT; β-actin-R, CAAACATGATCTGGGTCATCTTCTC.

### 4.7. Luciferase Reporter Assay

The transcriptional activity of the HIF transcription factor was measured as previously described [64]. A pGL2 luciferase reporter construct containing three HREs from the phosphoglycerate kinase 1 (PGK1) promoter (pGL2-PGK1-HRE-Luc) was purchased from Addgene (26731, Cambridge, MA, USA) and used to detect HIF transcriptional activity. Cells were co-transfected with pGL2-PGK1-HRE-Luc and pCMV-β-galactosidase. Forty-two hours later, the cells were lysed with luciferase cell lysis buffer (25 mM Gly-Gly (pH 7.8), 15 mM MgSO_4_-7H_2_O, 4 mM EGTA (pH 8.0), 1% Triton X-100 and 1 mM DTT). Luciferase and β-galactosidase activity was measured using luciferin and *O*-nitrophenyl-β-d-galactopyranoside, respectively, as substrates. Transfection efficiency was normalized to β-galactosidase activity.

### 4.8. Statistical Analysis

All experiments were done more than three times. The expression levels of each protein and mRNA were quantified by densitometry using ImageJ software. The expression level of each protein and mRNA was normalized against that of loading control. All bars are expressed as means ± standard error of mean (SEM). Unpaired two-tailed *t*-test was used for statistical analysis and statistical difference represented as asterisks (*) was considered significant when *p* < 0.05.

## Abbreviations

ARNT	Aryl hydrocarbon receptor nuclear translocator
BA	Bafilomycin A1
CCAR2	Cell cycle and apoptosis regulator 2
DMOG	Dimethyloxaloylglycine
HIF	Hypoxia-inducible factor
HRE	Hypoxia response elements
PCAF	p300/CBP-associated factor
PHD	Prolyl-4-hydroxylase domain-containing proteins
PPM1G	Mg^2+^/Mn^2+^-dependent protein phosphatase 1 gamma
VHL	Von Hippel-Lindau
